# Supplementary material for: Identification of photoperiod-induced specific miRNAs in the adrenal glands of Sunite sheep (Ovis aries)
Source: Front Vet Sci. 2022 Jul 22;9:888207. doi: 10.3389/fvets.2022.888207 (PMC9354845; doi:10.3389/fvets.2022.888207)
Supplement: Supplementary file 1 [file Data_Sheet_1.ZIP › Supplementary file/miRDeep2 file.zip!/miRDeep2 file/Know_Structure/SPLP42A3/oar-mir-487a.pdf]

miRBase precursor : oar-mir-487a
Total read count : 84
oar-miR-487a-5p read count : 16
oar-miR-487a-3p read count : 68
remaining reads : 0

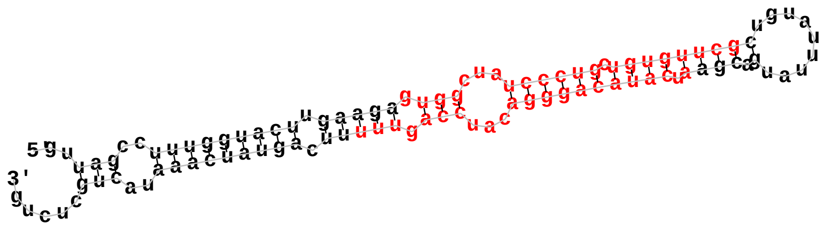

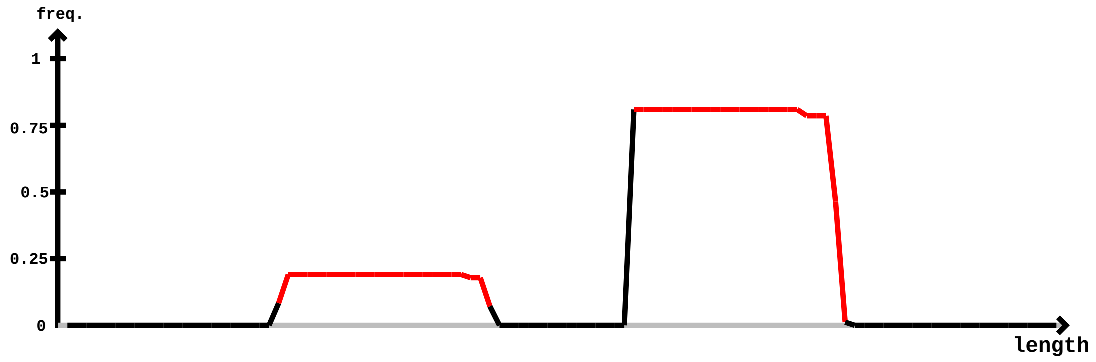


```
                                                       oar-miR-487a-3p
                    oar-miR-487a-5p
5'- guuagccuuugguacuugaagaguggcuaucccugcuguguucgcuguauuuaugacgaaucauacagggacauccaguuuuucaguaucaaauacugcucug   -3'    exp
    ..(((..(((((((((.(((((.(((...((((((.(((((((((.........).)))).))))))))))...))).))))).)))))))))..))).....   reads    mm    sample
    .....................aguggcuaucccugcugugu..............................................................   1        0     seq
    .....................aguggcuaucccugcuguguuc............................................................   6        0     seq
    ......................guggcuaucccugcuguguuc............................................................   3        0     seq
    ......................guggcuaucccugcuguguucg...........................................................   6        0     seq
    ..........................................................aaucauacagggacaucc...........................   2        0     seq
    ..........................................................aaucauacagggacauccagu........................   27       0     seq
    ..........................................................aaucauacagggacauccaguu.......................   38       0     seq
    ..........................................................aaucauacagggacauccaguuu......................   1        0     seq
```
